# Effects of ultra-high-pressure annealing on characteristics of vacancies in Mg-implanted GaN studied using a monoenergetic positron beam

**DOI:** 10.1038/s41598-020-74362-9

**Published:** 2020-10-15

**Authors:** Akira Uedono, Hideki Sakurai, Tetsuo Narita, Kacper Sierakowski, Michal Bockowski, Jun Suda, Shoji Ishibashi, Shigefusa F. Chichibu, Tetsu Kachi

**Affiliations:** 1 Division of Applied Physics, Faculty of Pure and Applied Science, University of Tsukuba, Tsukuba, Ibaraki 305-8573 Japan; 2 IMaSS, Nagoya University, Aichi, 464-8601 Japan; 3 Department of Electronics, Graduate School of Engineering, Nagoya University, Aichi, 464-8601 Japan; 4 ISET, ULVAC, Inc., Chigasaki, Kanagawa 253-8543 Japan; 5 Toyota Central R&D Labs., Inc., Nagakute, Aichi 480-1192 Japan; 6 Institute of High Pressure Physics, Polish Academy of Sciences, Sokolowska 29/37, 01-142 Warsaw, Poland; 7 Research Center for Computational Design of Advanced Functional Materials (CD-FMat), National Institute of Advanced Industrial Science and Technology (AIST), Tsukuba, Ibaraki 305-8568 Japan; 8 Institute of Multidisciplinary Research for Advanced Materials, Tohoku University, Sendai, 980-8577 Japan

**Keywords:** Engineering, Materials science

## Abstract

Vacancy-type defects in Mg-implanted GaN were probed by using a monoenergetic positron beam. Mg ions were implanted into GaN to obtain 0.3-μm-deep box profiles with Mg concentrations of 1 × 10^19^ cm^−3^. The major defect species in an as-implanted sample was determined to be Ga-vacancy related defects such as a complex between Ga and N vacancies. The sample was annealed under a nitrogen pressure of 1 GPa in a temperature range of 1000–1480 °C without a protective capping layer. Compared with the results for Mg-implanted GaN annealed with an AlN capping layer, the defect concentration was decreased by the cap-less annealing, suggesting that the surface of the sample was an effective sink for vacancies migrating toward the surface. Depth distributions of Mg after annealing above 1300 °C were influenced by the presence of residual vacancies at this temperature. Hydrogen atoms were unintentionally incorporated into the sample during annealing, and their diffusion properties were also affected by both vacancies and Mg.

## Introduction

Gallium nitride (GaN) based power devices have attracted worldwide attention because of its excellent physical properties such as their wide bandgap, high saturation electron velocity, sufficient thermal conductivity, and high breakdown voltage^1–3^. Lateral power transistors and diodes based on AlGaN/GaN heterostructures are utilized widely in RF and power conversion applications. Due to their structures, however, the chip size tends to increase as the power rating increases, which often degrades device properties and reliability. In addition, the stability of the device performance is known to be affected by trapping and/or scattering centers of carriers at the AlGaN surface and/or near the dielectric/AlGaN interface. Vertical GaN power devices have attracted significant attention because the breakdown voltage can be increased as the thickness of the drift region increases without enlarging the chip size^3,4^. This structure also leads to superior reliability due to reduction in the high electric field from the surface.

These vertical power devices require embedded p-type regions to suppress the electric field spreading near the edges of junctions, which is effective for obtaining high breakdown voltages^4^. Ion implantation is a common technique for selective p-type doping. The effects of ion implantation into GaN have been widely studied, and it is well known that the removal of implantation induced defects is difficult compared with that for Si or SiC^5,6^. During annealing, implantation induced point defects can either recombine or interact to form various kinds of defects or extended defects such as stacking faults and dislocation loops. The annealing temperature of such defects was reported to higher than the decomposition temperature of GaN (about 850 °C, Ref.^7^). However, several reports of successful Mg activation have started to appear recently^8–13^. In order to suppress surface damages during high-temperature annealing, a protective capping layer is deposited on the surface of GaN. Here, the chemical stability of the interface between the capping layer and GaN is important. Jacobs et al.^13^ reported that surface degradation due to multicycle rapid thermal annealing depended on the selection of materials and/or the deposition process of the capping layer. Narita et al.^10^ reported p–n diode formation by Mg- and H-implantation, where the ions were implanted into GaN(000$\stackrel{-}{1}$) substrates. Since the (000$\stackrel{-}{1}$) face is thermally more stable than the (0001) face, the annealing (1230 °C) was performed without a capping layer. Recently, Sakurai et al.^11^ reported that a high activation rate for Mg (70%) can be obtained without a capping layer by using an ultra-high-pressure annealing (UHPA) process. In their experiments, annealing was done with an N_2_ pressure of 1 GPa, and the maximum temperature was 1480 °C.

The materials and deposition processes of the capping layer are designed to suppress interatomic diffusion between the capping layer and GaN during high-temperature annealing. But this property also suppresses the migration of vacancy-type defects toward the surface and results in vacancy clustering below the capping layer^14^. Although an annealing treatment without a capping layer is ideal, the behaviors of point defects during annealing are not known. It is known that nitrogen vacancy (*V*_N_) acts as a compensator^15^, but other defects, such as complexes between Mg and vacancies, are also candidates which decrease an activation rate of Mg. Since many kinds of defects are introduced by ion implantation and post-implantation annealing^6^, a study of annealing behaviors of defects, such as agglomeration of vacancies and their dissociation, is also important for effectively utilizing ion implantation for GaN devices. Positron annihilation is a useful technique for a study of vacancy-type defects in semiconductors^16,17^, and this technique has been successfully used to detect vacancy-type defects in GaN^18–21^. In the present study, we used a monoenergetic positron beam to study the annealing behaviors of vacancy-type defects in Mg-implanted GaN with UHPA.

## Experiment

The present samples were undoped GaN films with 2-µm-thickness grown by using metal–organic vapor phase deposition (MOVPE). Details on the sample preparation process and the electric properties of the samples after UHPA are given elsewhere^11,12^. Before the deposition of the top GaN film, a 0.2-μm-thick buffer layer (n^+^-GaN) was grown on *c*+-GaN substrates obtained by hydride vapor phase epitaxy (HVPE). Carbon and oxygen concentrations in the GaN film were estimated by secondary ion mass spectrometry (SIMS), and they were lower than 4 × 10^15^ cm^−3^ and 3 × 10^15^ cm^−3^, respectively (detection limit). Mg^+^ ions were implanted into the samples to obtain 300-nm-deep box profiles with Mg concentrations [Mg] of 1 × 10^19^ cm^−3^ at room temperature. The energies of Mg ions were 30, 70, 150, and 300 keV, and the corresponding dosages were 2.0 × 10^13^, 5.0 × 10^13^, 1.1 × 10^14^, and 3.0 × 10^14^ cm^−2^, respectively. The samples were annealed at temperatures between 1000 and 1480 °C (5 min) under a N_2_ pressure of 1 GPa using a high-nitrogen-pressure solution system^22^, where the heating and cooling rates were 27 and 200 °C/min, respectively.

Details on the positron annihilation technique are described elsewhere^14,20,23^. In the present experiments, the Doppler broadening spectra of the annihilation radiation were measured with high purity Ge detectors as a function of the incident positron energy *E*. The obtained spectra were characterized by the *S* parameter, defined as the fraction of annihilation events over the energy range of 510.22‒511.78 keV, and by the *W* parameter, defined as the number of annihilation events in the energy ranges of 504.14‒507.96 keV and 514.04‒517.86 keV. The energy resolution of the Ge detectors was 1.2 keV (full-width at half-maximum: FWHM). Doppler broadening spectra were also measured with a coincidence system^16^ in darkness and while the samples were illuminated with a He–Cd laser (wavelength: 325 nm). The obtained *S*‒*E* curves were analyzed by VEPFIT, a computer program developed by van Veen et al.^24^. In the analysis, for the density of the sample, we used a value of 6.15 g/cm^3^. The application of the VEPFIT code to ion implanted GaN is described elsewhere^14,20,23^. Doppler broadening spectra corresponding to a delocalized positron and to positrons trapped by typical cation vacancies were calculated using the QMAS (Quantum MAterials Simulator) code^25^. An orthorhombic supercell equivalent to 4 × 4 × 2 wurtzite cells was used for simulation and it contained 128 atoms if there are no vacancies. The supercell dimensions were $2\sqrt 3$ *a*_0_ × 4 *a*_0_ × 2 *c*_0_, where *a*_0_ = 0.3189 nm and *c*_0_ = 0.5186 nm were the experimental lattice parameters of the wurtzite cell. For the supercell containing a defect, atomic positions in the fixed cell were computationally optimized through a series of first-principles calculations. Charge states of the system were assumed to be neutral. Additional information is given in supplementary document and further details on the calculation procedure are given elsewhere^25,26^.

## Results and discussion

**Figure 1:**
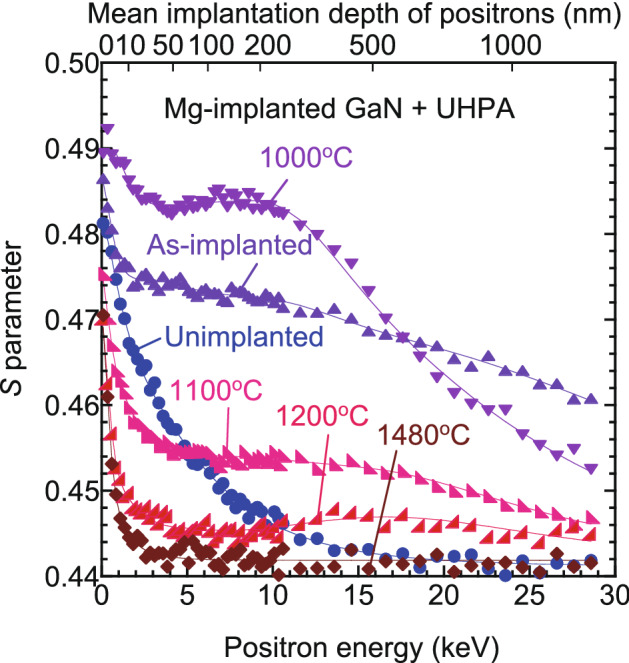
*S* parameter as function of incident positron energy *E* for Mg-implanted GaN before and after ultra-high-pressure annealing (UHPA). *S*‒*E* curve for unimplanted sample is also shown. Annealing temperatures are shown in figure. Solid curves are fits to experimental data.

Figure 1 shows the values of *S* parameter of Mg-implanted GaN before and after UHPA as a function of incident positron energy *E*. The *S*‒*E* curve for an unimplanted sample is also shown. The mean implantation depth of positrons is shown on the upper horizontal axis. For unimplanted GaN, the large *S* value was observed near the surface (*E* ≅ 0.1 keV) and it decreased with decreasing *E*. This is due to the annihilation of positrons at the surface and the decrease in the annihilation probability at the surface at high *E*. From the fitting, the diffusion length of positrons *L*_d_ was obtained as 110 ± 3 nm, which corresponds to the one for high-quality GaN^14,23^. The *S* value saturated at *E* > 20 keV, suggesting that almost all positrons annihilated in the bulk in this energy range, and this value was close to the *S* value for defect-free GaN^14,20,23^. For the sample annealed at 1480 °C, the *S* value saturated at *E* > 2 keV, suggesting that the effect of the positron annihilation at the surface was negligible at this energy range. The saturated *S* value was close to the *S* value for defect-free GaN^14,20,23^. The observed *S*–*E* curve for the sample annealed at 1480 °C was close to that for Mg-doped GaN grown by MOVPE^27^, suggesting a high activation rate of Mg. For the as-implanted sample and ones annealed at 1000–1200 °C, the *S* values were larger than *S* for defect-free GaN, which can be attributed to the trapping of positrons by vacancy-type defects.

**Figure 2:**
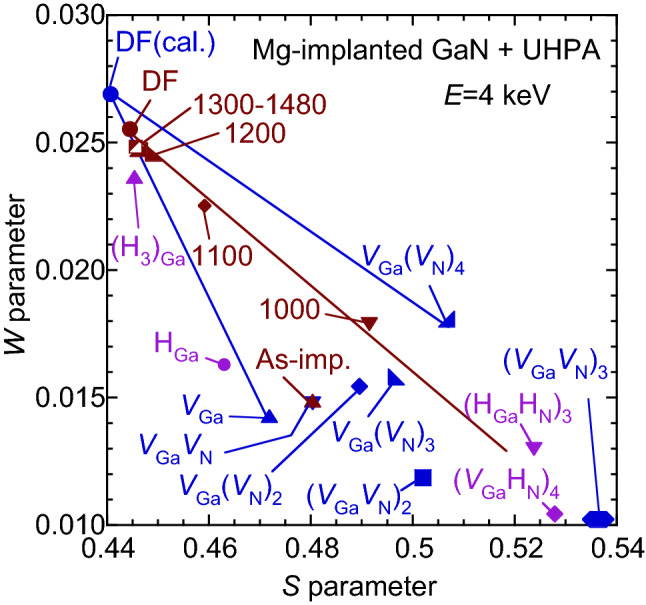
(*S*, *W*) values measured at *E* = 4 keV using coincidence system (brown symbols). (*S*, *W*) for unimplanted GaN is also shown (denoted as defect-free; “DF”). Calculated (*S*, *W*) for positron annihilation in delocalized state, DF, *V*_Ga_, *V*_Ga_(*V*_N_)_n_, (*V*_Ga_*V*_N_)_m_, and complexes between vacancies and H [H_Ga_, (H_3_)_Ga_, (H_Ga_H_N_)_3_, and (*V*_Ga_H_N_)_4_] are also shown (blue symbols), where notation “(H_n_)_Ga_” corresponds to *V*_Ga_ containing H atoms, and n is number of H atoms in *V*_Ga_. Brown line connects (*S*, *W*) values for unimplanted sample and samples annealed above 1000 °C.

From the relationship between the *S* and *W* values, the defect species detected by positron annihilation can be identified^16^. Figure 2 shows the (*S*, *W*) values measured by using the coincidence system at *E* = 4 keV for the Mg-implanted GaN before and after annealing. Almost all positrons with this energy were implanted into the region corresponding to the box profile of Mg. The statistical error of the (*S*, *W*) values was close to the size of symbols used in the figure. The (*S*, *W*) value for unimplanted GaN was measured at *E* = 28.6 keV, and it was also shown to be the value for the defect-free GaN (denoted as “DF”). The (*S*, *W*) values calculated for the annihilation of positrons in the delocalized state [DF(cal.)], typical complexes of Ga-vacancies (*V*_Ga_) and *V*_N_, such as (*V*_Ga_)_m_(*V*_N_)_n_, and complexes between vacancies and H are shown (blue symbols). In the figure, the notation “(H_n_)_Ga_” corresponds to *V*_Ga_ containing H atoms, and n is number of H atoms in *V*_Ga_. “(H_Ga_H_N_)_3_” means that one H atom is trapped by *V*_Ga_ and *V*_N_, respectively, and each of them forms six-fold complexes. The difference between the (*S*, *W*) value for the unimplanted GaN (DF) and the calculated one for defect-free GaN [DF(cal.)] is due to a limitation of the simulation used in the present work. For the as-implanted sample, the (*S*, *W*) value was close to the calculated value for *V*_Ga_*V*_N_. Because almost all positrons annihilated in the trapped state in the damaged region for as-implanted GaN^14,20,23^, the major defect species in this sample can be identified as *V*_Ga_-related defects such as *V*_Ga_*V*_N_. The same conclusion was reached in previous reports^14,20,23,28^. After annealing at 1000–1480 °C, the (*S*, *W*) values located on the line connecting the values for DF-GaN and (*V*_Ga_*V*_N_)_3_. This suggests that vacancy clusters such as (*V*_Ga_*V*_N_)_3_ were introduced at 1000 °C annealing, and their effect on the positron annihilation parameters decreased above 1100 °C. The introduction of the vacancy clusters in the Mg-implanted GaN after annealing without the capping layer was also confirmed in measurements of the positron lifetime spectra^23^, and the observed annealing behavior of the defects was the same as that for the sample annealed without the capping layer. As mentioned below, a certain amount of H was incorporated in the present sample, and H atoms were considered to be trapped by vacancy-type defects. As shown in Fig. 2, because the *S* (*W*) value tended to be decreased (increased) by the trapping of H by vacancies, the size of the vacancies for the sample annealed at 1000 °C could be larger than that of (*V*_Ga_*V*_N_)_3_.

**Figure 3:**
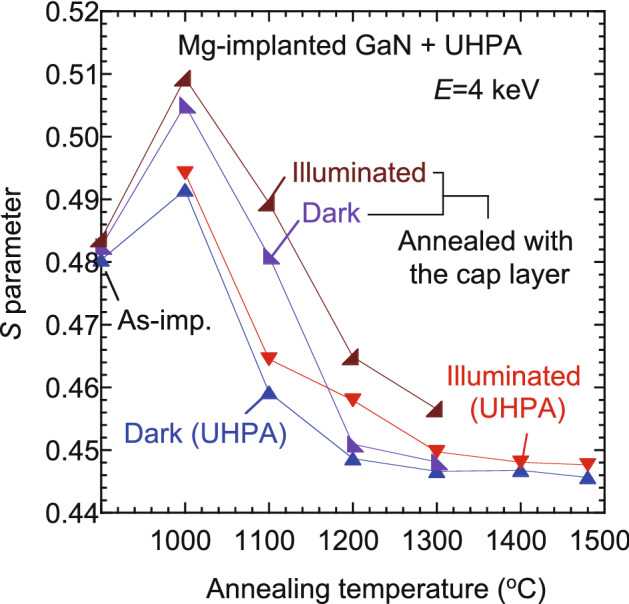
Annealing behaviors of *S* for Mg-implanted GaN before and after UHPA. Results for Mg-implanted GaN annealed with protective capping layer are also shown^23^.

Figure 3 shows the annealing behaviors of the *S* values calculated from the coincidence Doppler broadening profiles measured at *E* = 4 keV for the Mg-implanted GaN before and after UHPA. Uedono et al.^23^ reported the results for Mg-implanted GaN annealed with a 300-nm-thick AlN capping layer in N_2_ gas at atmospheric pressure (1000–1300 °C). The obtained annealing behaviors of the *S* values for those samples are also shown. For the sample annealed with the capping layer, Mg^+^ ions were implanted into the sample to obtain 500-nm-box profiles with [Mg] = 1 × 10^19^ cm^−3^ at room temperature. The defect species in the sample before annealing was identified as *V*_Ga_-related defects, which was the same as that for the present sample. No large difference in overall annealing behaviors of *S* measured in darkness was observed for the samples with and without the capping layer. As discussed above, the increase in the *S* value for the sample annealed at 1000 °C corresponds to the formation of vacancy clusters. At 1000–1300 °C, the *S* values for the sample annealed with the capping layer were larger than those for the sample with UHPA, suggesting that the annealing without a capping layer effectively decreased the concentration of vacancy-type defects even at a relatively low annealing temperature (1000 °C).

The trapping rate of positrons by vacancy-type defects depends on the defect charge state^16^. In general, when defects with different charge states coexist in a sample, the trapping of positrons by positively charged defects is negligible. Thus, the observed decrease in the *S* value for the samples annealed above 1100 °C was attributed to not only a decrease in the size/concentration of vacancy-type defects but also to the downwards shift of the Fermi level position due to Mg activation and a resultant shift of the defect charge state from neutral to positive (or negative to neutral)^20,23^. The *S* values for both samples were increased by illumination, which can be attributed to the transition in the charge state of the vacancy-type defects from positive to neutral (or neutral to negative) and a resultant increase in the trapping probability of positrons to such defects^20,23,29^. The effect of the illumination on the *S* value for the sample annealed with the capping layer was larger than that for the sample with UHPA, which suggests that the concentration of residual vacancies, which act as acceptors, was high for the sample annealed with the capping layer. This also shows the advantage of the annealing treatment without a capping layer.

**Figure 4:**
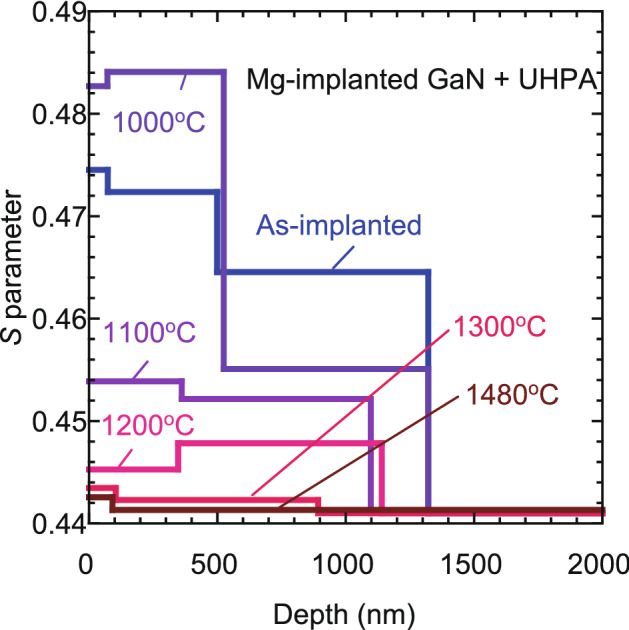
Depth distributions of *S* obtained from analysis of *S*–*E* curves for Mg-implanted GaN (Fig. 1).

**Figure 5:**
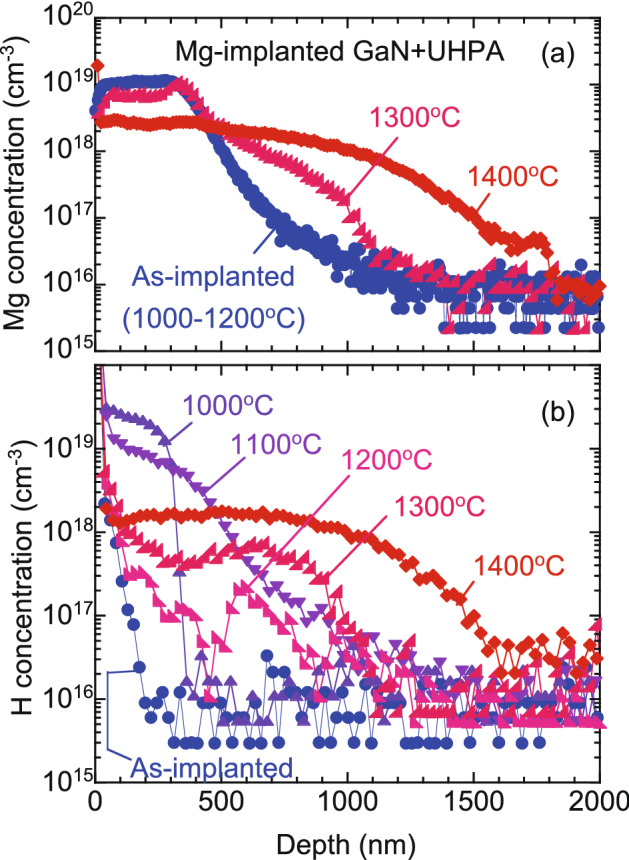
Depth distributions of Mg and H for Mg-implanted GaN measured by SIMS.

The solid curves in Fig. 1 are fits to the experimental data. In the fitting, the region sampled by positrons was divided into three or four blocks. Derived depth distributions of *S* are shown in Fig. 4. The *L*_d_ value in the first block (the nearest surface region) was determined by the fitting, and the values for the samples annealed at different temperatures were obtained to be 5‒10 nm, which were the typical for materials with high vacancy concentration^14,16^. The *L*_d_ values in other blocks corresponding to the damaged region were fixed to be 10 nm. These values did not significantly influence the obtained depth profiles of *S*. Figure 5a,b shows the depth distributions of Mg and H measured by SIMS. The incorporation of H in the samples during UHPA was reported by Narita et al.^12^. They suggested that unintentional moisture may exist in the UHPA chamber, and this caused doping of H into the sample during UHPA. No difference in the Mg distributions for the as-implanted sample and the samples annealed at 1000‒1200 °C was observed. After annealing at 1300 °C, Mg started to diffuse toward the inside of the samples. The Mg distribution for the sample annealed at 1400 °C was identical to that for the sample annealed at 1480 °C. In Fig. 4, for the as-implanted sample and the sample annealed at 1100 °C, the position of the first and second blocks with a high *S* value (≤ 500 nm) agreed with the box profile of Mg. This can be attributed to the high [Mg] in this region and a resultant high total deposited energy of Mg that was used to replace atoms. In Fig. 4, a damaged region was introduced up to a depth 900‒1300 nm, and this region was deeper than the block profile of Mg. This was due to the introduction of defects by Mg implanted below 300 nm (which can be seen as a tail from the Mg block profile) and the high sensitivity of positrons to vacancy-type defects.

In Fig. 5b, for the as-implanted sample, the observed high concentration of H near the subsurface (≤ 200 nm) was due to surface adsorbates. The incorporation of H was observed after annealing at 1000 °C, where the H concentration was high in the region corresponding to the Mg box profile (≤ 300 nm), and it rapidly decreased below 300 nm. The observed box profile of H is unlikely explainable assuming the diffusion model with a constant source at the surface. It is well known that H incorporation is often caused by Mg-doped GaN because of the low formation energy of Mg_Ga_-H pairs^12,30^. For the sample annealed at 1000 °C, vacancy-type defects with a high concentration still remained, and as can be seen in Fig. 5, the concentration of H was higher than that of Mg in this region. Thus, the major cause of the box-shape H distribution could be attributed to the trapping of vacancy-type defects. After 1100 °C annealing, H atoms started to diffuse toward the bulk, and as a result, the H concentration in the subsurface region decreased. A further decrease in the H concentration was observed in the subsurface region after annealing at 1200 °C. As mentioned above, because the surface acted as a sink for vacancies even in this temperature range, the observed change in the H concentration in the subsurface region could be related to the diffusion of vacancy-type defects toward the surface. For this sample, the H concentration in the region between 500 and 900 nm remained high, and this region reasonably agreed with the second block (400–1100 nm) in the depth distribution of *S* (Fig. 4). This can be attributed to the trapping of H by vacancy-type defects in the deep defect rich region and to a resultant suppression of the out-diffusion of H toward the surface. Because no large change in the depth distribution of Mg was observed up to this annealing temperature (1200 °C), the observed annealing behavior of H did not directly relate to the interaction between Mg and H.

After annealing at 1300 °C, Mg started to diffuse toward the bulk, but it seemed to be suppressed below the deeper defect rich region (400–1100 nm), suggesting that the depth distribution of Mg was influenced by the presence of vacancy-type defects. The H concentration started to increase at this annealing temperature, which was associated with the enhanced incorporation of H during the annealing. Above 1400 °C annealing, both the depth distribution and the concentration of Mg almost coincided with those of H, respectively. In this temperature range, because of the decrease in the vacancy concentration (Fig. 4), the number of Mg atoms at the Ga sites increased, and as a result, the interaction between Mg_Ga_ and H was likely to enhance the incorporation of H in the sample.

## Summary

The positron annihilation technique was used to study behaviors of vacancy-type defects in Mg-implanted GaN annealed without a protective capping layer. The major defect species in the as-implanted GaN was identified as *V*_Ga_-related defects such as *V*_Ga_*V*_N_. Compared with the results for a sample with a capping layer, the vacancy concentration was decreased by UHPA without the use of a capping layer. This suggests that the surface is an effective sink for vacancy-type defects, and it also shows the superiority of high-temperature annealing with a non-capping layer. The annealing behaviors of vacancies related to the change in the depth distributions of Mg and H, where H was unintentionally incorporated in the sample during UHPA. In the temperature range between 1000 and 1200 °C, no change in the depth distribution of Mg was observed. The depth distribution of H, however, was influenced by vacancy distributions, which was due to the trapping of H by such defects. Above 1300 °C annealing, Mg atoms started to diffuse toward the bulk. In this temperature range, H incorporation was mainly caused by the interaction between Mg_Ga_ and H. The present research shows that the vacancy-type defects and their annealing behaviors affected the depth distribution of Mg during annealing. Knowledge on the interaction between vacancies, Mg, and H is useful for optimizing the p-type doping process using ion-implantation.

## Supplementary information

Supplementary Information.
