# Supplementary material for: Effects of ultra-high-pressure annealing on characteristics of vacancies in Mg-implanted GaN studied using a monoenergetic positron beam
Source: Sci Rep. 2020 Oct 15;10:17349. doi: 10.1038/s41598-020-74362-9 (PMC7562725; doi:10.1038/s41598-020-74362-9)
Supplement: Supplementary file 1 — Supplementary Information. [file 41598_2020_74362_MOESM1_ESM.pdf]

Effects of ultra-high-pressure annealing on characteristics of vacancies in Mg-implanted GaN studied using a monoenergetic positron beam

Akira Uedono,[1,a)] Hideki Sakurai,[2,3,4] Tetsuo Narita,[5] Kacper Sierakowski,[6] Michal Bockowski,[2,6] Jun Suda,[2,3] Shoji Ishibashi,[7] Shigefusa F. Chichibu,[2,8] and Tetsu Kachi[2]

[1]*Division of Applied Physics, Faculty of Pure and Applied Science, University of Tsukuba, Tsukuba, Ibaraki 305-8573, Japan*
[2]*IMaSS, Nagoya University, Aichi 464-8601, Japan*
[3]*Department of Electronics, Graduate School of Engineering, Nagoya University, Aichi 464-8601, Japan*
[4]*ISET, ULVAC, Inc., Chigasaki, Kanagawa 253-8543, Japan*
[5]*Toyota Central R&D Labs., Inc., Nagakute, Aichi 480-1192, Japan*
[6]*Institute of High Pressure Physics, Polish Academy of Sciences, Sokolowska 29/37, 01-142 Warsaw, Poland*
[7]*Research Center for Computational Design of Advanced Functional Materials (CD-FMat), National Institute of Advanced Industrial Science and Technology (AIST), Tsukuba, Ibaraki 305-8568, Japan*
[8]*Institute of Multidisciplinary Research for Advanced Materials, Tohoku University, Sendai 980-8577, Japan*

*corresponding author: uedono.akira.gb@u.tsukuba.ac.jp

1. Computer simulation of implantation profiles of Mg in GaN

Figures 1 and 2 shows the depth distribution of Mg for the as-implanted sample. The results obtained by TCAD Sentaurus device simulator [1] and SRIM-2013 code [2] were also shown in Figs. 1 and 2, respectively. Figure 3 shows the depth distributions of energy transferred from ion to recoil atoms obtained by SRIM-2013. The implantation energies of Mg (30, 70, 150, and 300 keV) are shown in these figures. Although an overall Mg profile was well represented by Sentaurus device simulator, a long tail of the Mg profile below 800 nm was not obtained. The difference between the depth profiles of Mg simulated by Sentaurus and SRIM increased as the energy of ions increased.

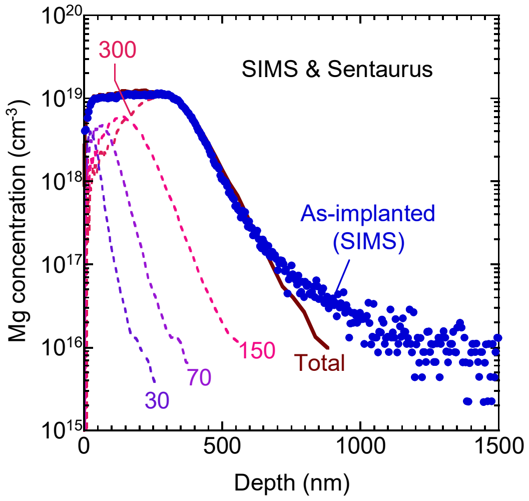

Fig. 1. Depth distributions of Mg for as-implanted GaN measured by SIMS. Results obtained by TCAD Sentaurus device simulator [1] are also shown. Energies of implanted ions (30, 70, 150, and 300 keV) are shown in the figure.

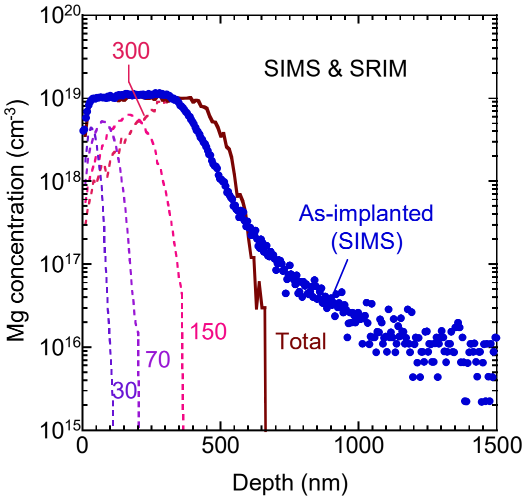

Fig. 2. Depth distributions of Mg for as-implanted GaN measured by SIMS. Results obtained by SRIM-2013 code [2] are also shown.

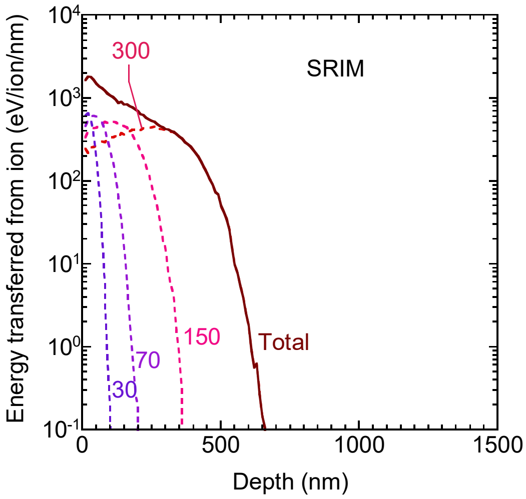

Fig. 3. Depth distributions of energy transferred from ion to recoil atoms obtained by SRIM-2013 code [2].

2. Theoretical calculation of positron states and annihilation parameters in GaN

The Doppler broadening spectra corresponding to positron annihilation in the delocalized state and trapped state in cation vacancies were calculated using QMAS (Quantum MAterials Simulator) code [3,4]. This program adopts the projector augmented-wave (PAW) method [5,6] to obtain electron wave functions. To describe the exchange and correlation energy of electrons, the generalized gradient approximation [7] was used. Details on the calculation procedure for group-III nitride semiconductors are given elsewhere [8]. For the high-frequency dielectric constant, which is used in evaluating the enhancement factor (Eq. 4 of Ref. 8), we used the experimental value of 5.5. An orthorhombic supercell equivalent to 4 × 4 × 2 wurtzite cells was used for simulation and it contained 128 atoms if there are no vacancies. The supercell dimensions were $2\sqrt{3}\ a_0 \times 4\ a_0 \times 2\ c_0$, where a_0 = 0.3189 nm and c_0 = 0.5186 nm were the experimental lattice parameters of the wurtzite cell. For the supercell containing a defect, atomic positions in the fixed cell were computationally optimized through a series of first-principles calculations. The planewave energy cutoff and the force convergence criterion are set to be 20 Ha and 5×10^{-5} Ha/bohr, respectively. Only the Γ point is used for the Brillouin zone sampling. Charge states of the system were assumed to be neutral. Effects of the trapping of positrons on the configuration of atoms were not included in the simulation. The formalism of the local density approximation [9] was used in the calculation of the positron wave functions. Doppler broadening spectra corresponding to the annihilation of positrons in the delocalized state and the trapped states in cation vacancies were calculated, and they were characterized by the S and W parameters.

3. S–W relationship for Mg-implanted GaN

Figure 4 shows the (S,W) values for the Mg-implanted samples with different annealing temperatures. The result for the unimplanted sample is also shown [Fig. 4(a)]. These

values were calculated from the Doppler broadening profile measured by using the conventional measurement system (non-coincidence mode). For the unimplanted sample, the (S,W) values measured at low E locate near (0.48,0.015), and the values shifted to the (S,W) value corresponding to the annihilation of positrons from the delocalized state (0.44,0.0275). This value was shown as an open red circle (denoted as DF). Almost all (S,W) values located on a line as shown in Fig. 1(a), but they tend to locate upper right from the line at E < 1.5 keV. This is mainly due to the increase in the annihilation probability of epithermal positrons at the surface. For the as-implanted sample [Fig. 4(b)], the (S,W) values shifted toward the right-hand side from the line, which is due to the annihilation of positrons in the damaged region introduced by Mg-implantation. The (S,W) values measured at E = 4 keV are shown as red circles. The (S,W) values calculated from the coincidence Doppler broadening profiles measured at E = 4 keV were shown in Fig. 3 in the paper. For the sample annealed at 1000°C [Fig. 3(c)], a further shift of the (S,W) value toward right-hand side was observed. With increasing annealing temperature [Fig. 3(d)–(f)], the (S,W) values tended approach the value for the defect-free state.

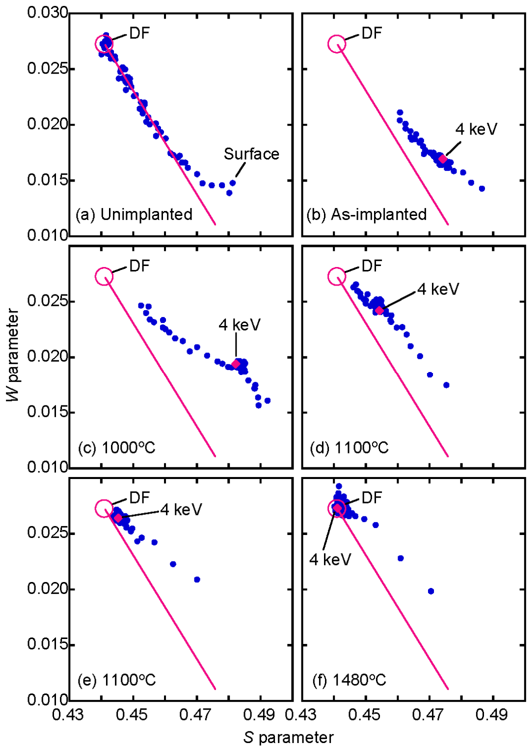

Fig. 4. (S,W) values for (a) the unimplanted sample and (b)-(f) Mg-implanted samples with different annealing temperature. Open red circles indicated as “DF” (defect free) show (S,W) values corresponding to annihilation of delocalized positron in GaN. Red circles show values measured at E = 4 keV.

References

1. Synopsys (2016). Sentaurus device user guide, version N-2017, 09. Mountain View

CA USA.

2. J. F. Ziegler, M. D. Ziegler, and J. P. Biersack, Nucl. Inst. Meth. B 268, 1818 (2010).
3. S. Ishibashi, T. Tamura, S. Tanaka, M. Kohyama and K. Terakura, Phys. Rev. B 76, 153310 (2007).
4. S. Ishibashi and A. Uedono, J. Phys. Conf. **505**, 012010 (2014).
5. P. E. Blöchl, Phys. Rev. B 50, 17953 (1994).
6. G. Kresse and D. Joubert, Phys. Rev. B **59**, 1758 (1999).
7. J. P. Perdew, K. Burke, and M. Ernzerhof, Phys. Rev. Lett. **77,** 3865 (1996).
8. S. Ishibashi, A. Uedono, H. Kino, T. Miyake, and K. Terakura, J. Phys. Cond. Matter **31**, 475401 (2019).
9. E. Boroński and R. M. Nieminen, Phys. Rev. B **34** , 3820 (1986).
